# Correction: Effects of extensive protein hydrolysate in supporting intestinal barrier function in vitro

**DOI:** 10.1038/s41390-026-04807-w

**Published:** 2026-02-06

**Authors:** Valentina Bozzetti, Rachel Brosnan, Smriti Verma, Tina Tran, Ruslan Sadreyev, Murat Cetinbas, Teresa Murguia- Peniche, Ruth Simmons, Gabriele Gross, Alessio Fasano

**Affiliations:** 1 Mucosal Immunology and Biology Research Center, Massachusetts General Hospital for Children, Boston, MA USA; 2 Experimental Gastroenterology Lab, Division of Immunology, Transplantation and Infectious Disease, Department of Gastroenterology and Digestive Endoscopy, IRCSS San Raffaele Hospital, Milan, Italy; 3 Department of Molecular Biology, Massachusetts General Hospital and Department of Pathology, Massachusetts General Hospital and Harvard Medical School, Boston, MA USA; 4 R&D Medical Sciences, Mead Johnson Nutrition Institute/Reckitt, Evansville, IN USA; 5 School of Medicine, Indiana University, Evansville, IN USA; 6 R&D Nutrition Science Platforms, Mead Johnson Nutrition Institute/Reckitt, Slough, UK; 7 R&D Nutrition Science Platforms, Mead Johnson Nutrition Institute/Reckitt, Nijmegen, The Netherlands

Correction to: *Pediatric Research* 10.1038/s41390-025-04680-z, published online 21 January 2026

In this article the author’s name Smriti Verma was incorrectly written as Smriti Verna. The original article has been corrected.

